# Supplementary material for: Improvement of contact lens-associated dry eye disease with the use of hydrogen peroxide
Source: PeerJ. 2024 Dec 6;12:e18482. doi: 10.7717/peerj.18482 (PMC11627073; doi:10.7717/peerj.18482)
Supplement: Supplemental Information 2 [file peerj-12-18482-s002.pdf]

Hoja de exploración del Proyecto

"Evaluación de actuaciones para la mejora del confort en el uso de lentes de contacto"

Nº IC: ___________ , Fecha: ______________ Sexo: _____________, Edad: ______________________
Años usando LC:_________________LC usada(s):____
Horas de uso/día: ________________ Sistema de limpieza: ____________________________________
Tiempo percibiendo incomodidad: ____
Optometrista: ____ Nº GO: _________________
Centro Óptico: ____ Nº CO: _________________

	AVcc	Refracción lentes de contacto	AVcc
**OD:**			
**OI:**			

Exploración Biomicroscópica

**Ojo Derecho**										**Ojo Izquierdo**
**0**	**1**	**2**	**3**	**4**		**0**	**1**	**2**	**3**	**4**
					**PÁRPADOS**					
					**Blefaritis**					
					**Meibomitis**					
					**CONJUNTIVA**					
					**Hiperemia Limbar**					
					**Hiperemia Bulbar**					
					**Conj. Tarsal Inf.**					
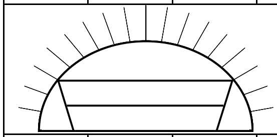			Total		**Conj. Tarsal Sup**	Total		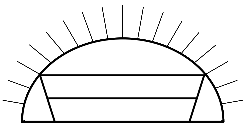		
					**Tinción Conjuntival**					
					**CÓRNEA**					
					**Edema**					
					**Infiltrados**					
					**Regularidad Endotelial**					
					**Vascularización corneal**					
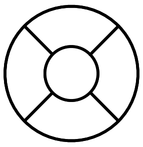			Total		**Tinción Fluoresceína**	Total		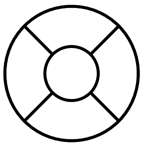		
					**LENTES CONTACTO**					
					**Humectabilidad LC**					
					**Depósitos superficie ant**					
					**Depósitos superficie post**					
					**Irregularidad superfic LC**					
					**Centrado**					
**-2**	**-1**	**0**	**+1**	**+2**	**Movimiento LC**	**-2**	**-1**	**0**	**+1**	**+2**

Observaciones:

centro inGO Proy. Piloto
investigar - innovar - inspirar...

OSDI: Índice de Trastorno de la Superficie Ocular

IC: Fecha

Observaciones

¿Ha experimentado alguno de los siguientes síntomas en la *última semana?*

	Constantemente	La mayor parte del tiempo	La mitad del tiempo	Ocasionalmente	Nunca
1. Ojos sensibles a la luz	4	3	2	1	0
2. Sensación de arena en los ojos	4	3	2	1	0
3. Dolor o irritación ocular	4	3	2	1	0
4. Visión borrosa	4	3	2	1	0
5. Visión reducida	4	3	2	1	0

Sumar los valores marcados como respuesta a las preguntas 1 a 5 (A)

¿Los problemas oculares le han dificultado alguna de las siguientes tareas en la *última semana?*

	Constantemente	La mayor parte del tiempo	La mitad del tiempo	Ocasionalmente	Nunca	N/S, N/C
6. Leer	4	3	2	1	0	0
7. Conducir de noche	4	3	2	1	0	0
8. Trabajar con ordenadores, usar un cajero automático	4	3	2	1	0	0
9. Ver la televisión	4	3	2	1	0	0

Sumar los valores marcados como respuesta a las preguntas 6 a 9 (B)

¿Ha sentido molestias oculares en alguna de las siguientes situaciones en la *última semana?*

	Constantemente	La mayor parte del tiempo	La mitad del tiempo	Ocasionalmente	Nunca	N/S, N/C
10. Viento	4	3	2	1	0	0
11. Zonas de humedad baja (muy secas)	4	3	2	1	0	0
12. Sitios con aire acondicionado	4	3	2	1	0	0

Sumar los valores marcados como respuesta a las preguntas 10 a 12 (C)

Puntuación OSDI		Grado de Severidad
**de 0 a 12**	→	**Normal**
**de 13 a 22**	→	**Medio**
**de 23 a 32**	→	**Moderado**
**de 33 a 100**	→	**Severo**

(D) Sume los resultados A, B y C para obtener D (D = suma de los resultados de todas las preguntas respondidas)

(E) Número total de preguntas respondidas (sin contar las respuestas N/S, N/C)

$$\text{Puntuación OSDI} = \frac{D \times 25}{E}$$
