# Supplementary material for: Improvement of contact lens-associated dry eye disease with the use of hydrogen peroxide
Source: PeerJ. 2024 Dec 6;12:e18482. doi: 10.7717/peerj.18482 (PMC11627073; doi:10.7717/peerj.18482)
Supplement: Supplemental Information 3 [file peerj-12-18482-s003.pdf]

EFRON GRADING SCALES FOR CONTACT LENS COMPLICATIONS

0 - NORMAL	1 - TRACE	2 - MILD	3 - MODERATE	4 - SEVERE

CONJUNCTIVAL REDNESS

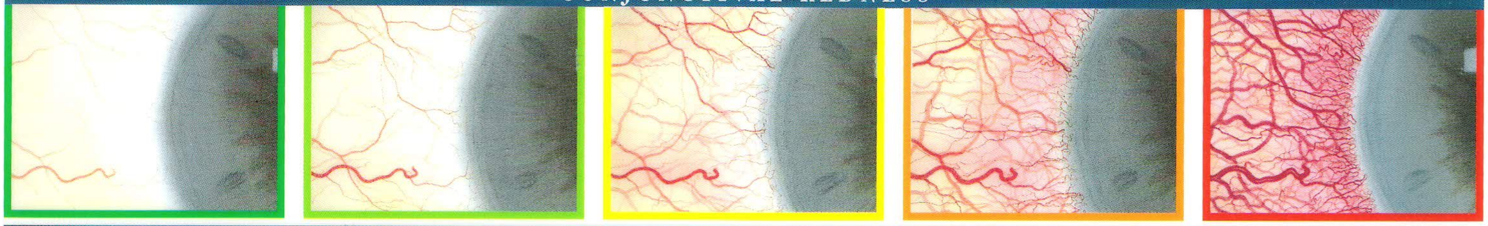

LIMBAL REDNESS

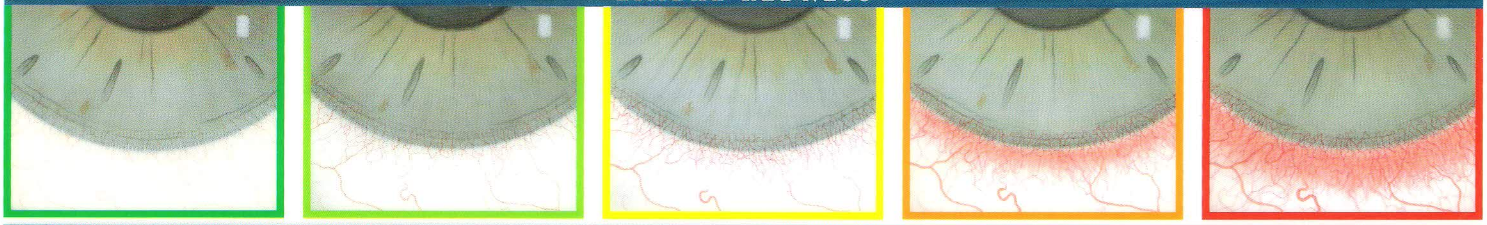

CORNEAL NEOVASCULARISATION

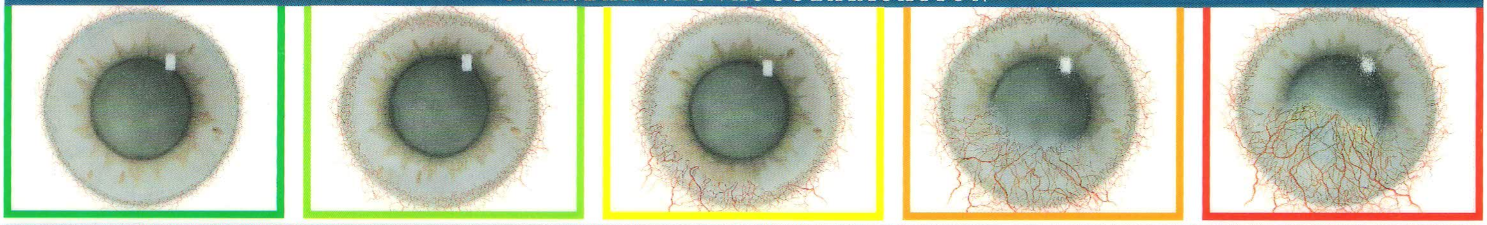

EPITHELIAL MICROCYSTS

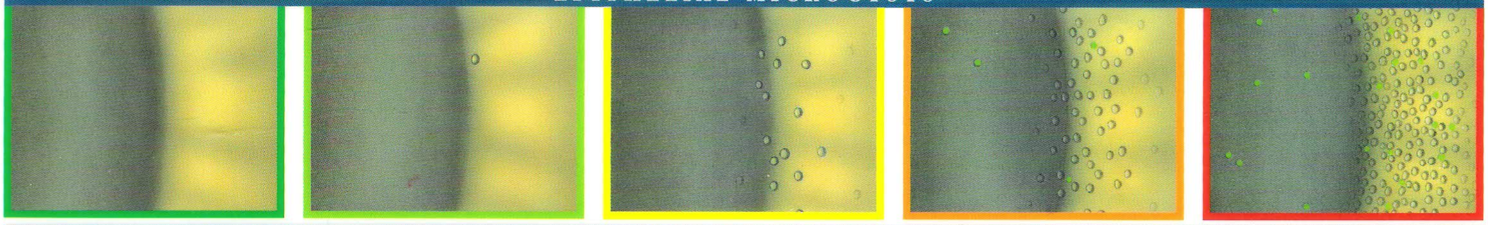

CORNEAL OEDEMA

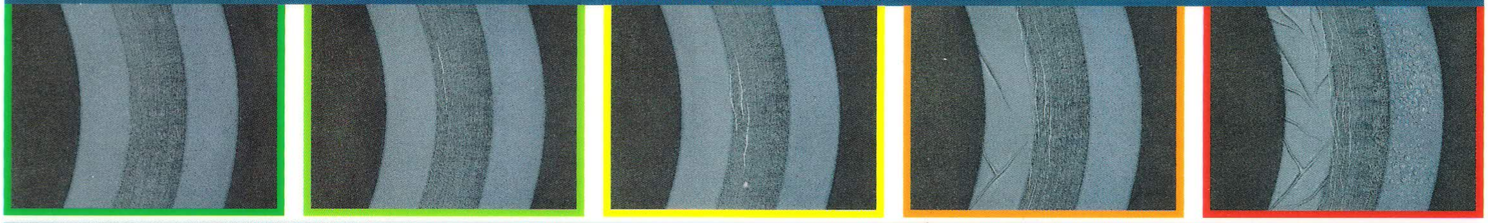

CORNEAL STAINING

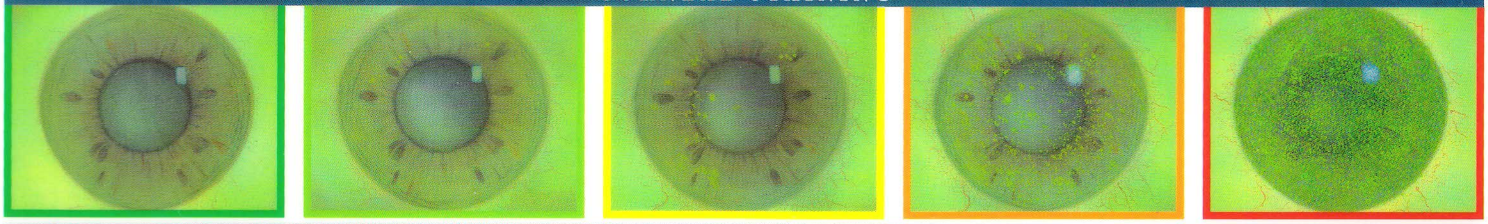

CONJUNCTIVAL STAINING

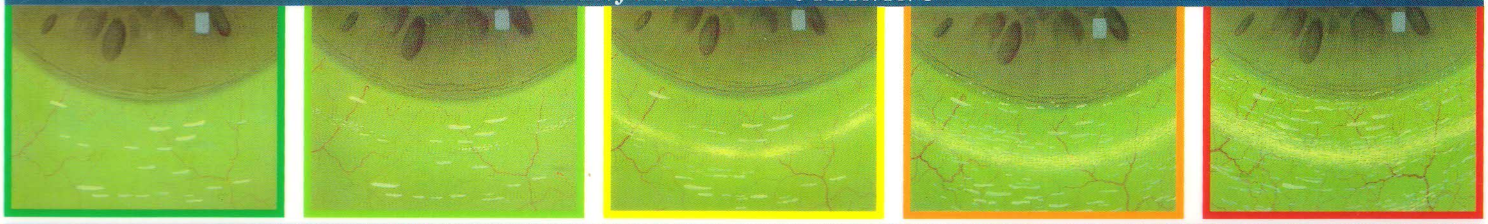

PAPILLARY CONJUNCTIVITIS

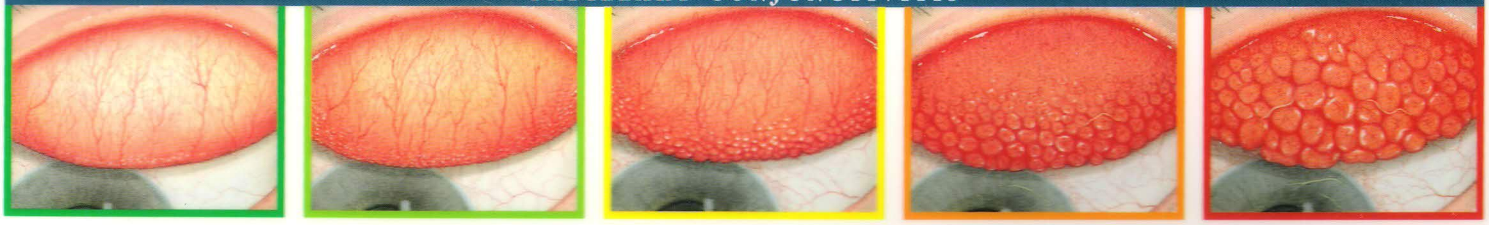

Devised by Professor Nathan Efron and illustrated by Terry R Tarrant. Millennium Edition. January 1, 2000
Supplement to the book *Contact Lens Complications* by N. Efron published by Butterworth-Heinemann/Optician, 1999, ISBN 0 7506 0582 0

Sponsored by HYDRON™

Efron Grading Scales for Contact Lens Complications

0 - NORMAL	1 - TRACE	2 - MILD	3 - MODERATE	4 - SEVERE

BLEPHARITIS

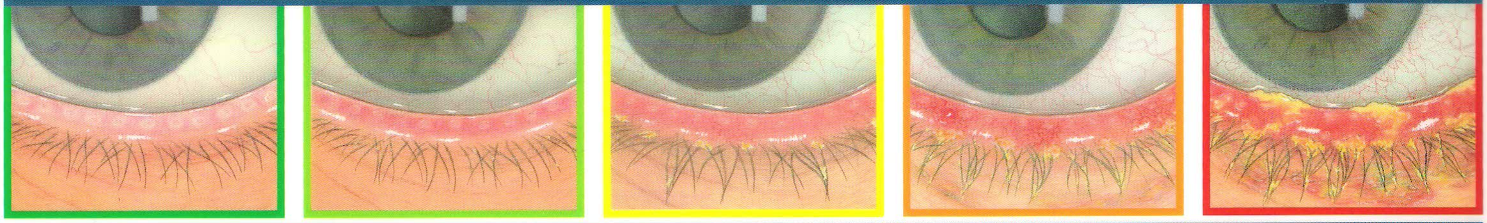

MEIBOMIAN GLAND DYSFUNCTION

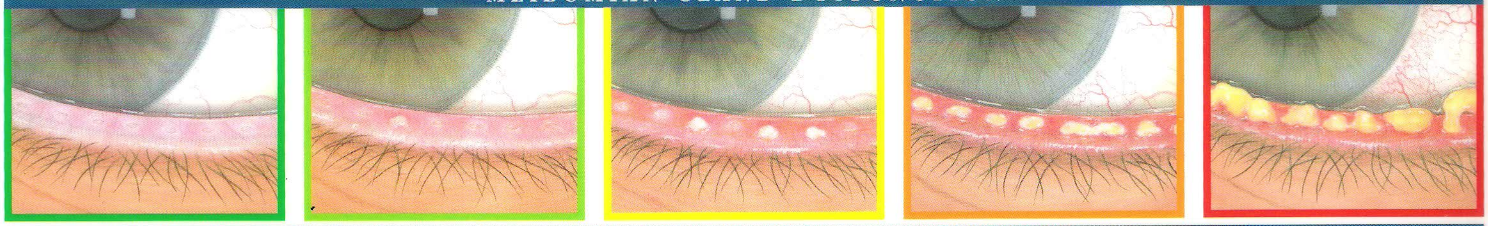

SUPERIOR LIMBIC KERATOCONJUNCTIVITIS

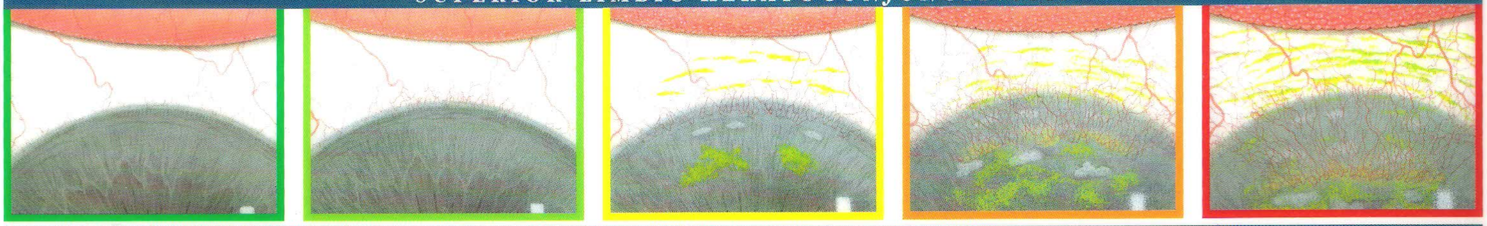

CORNEAL INFILTRATES

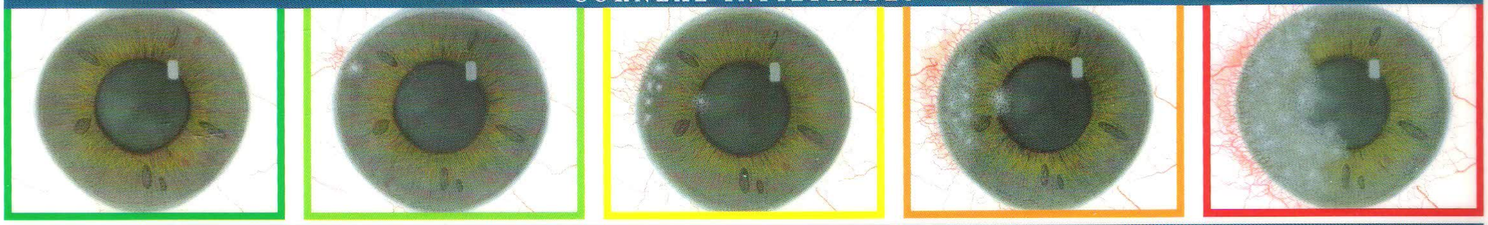

CORNEAL ULCER

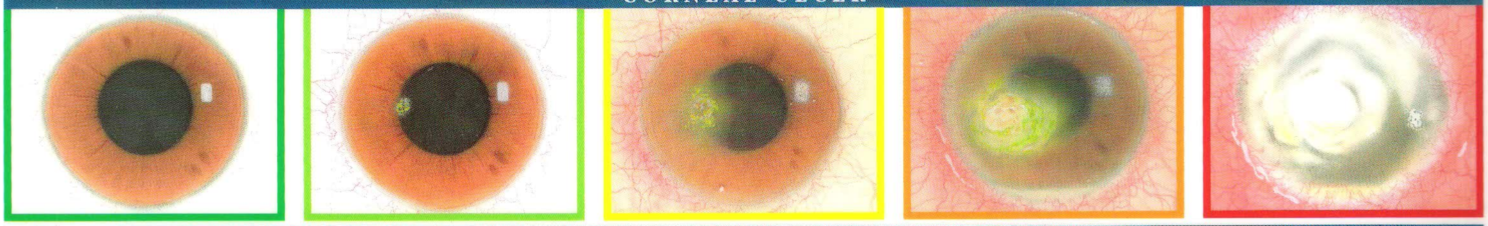

ENDOTHELIAL POLYMEGETHISM

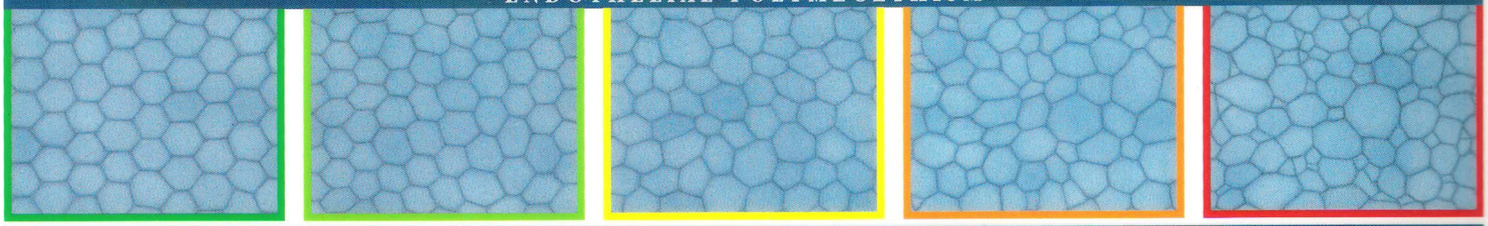

ENDOTHELIAL BLEBS

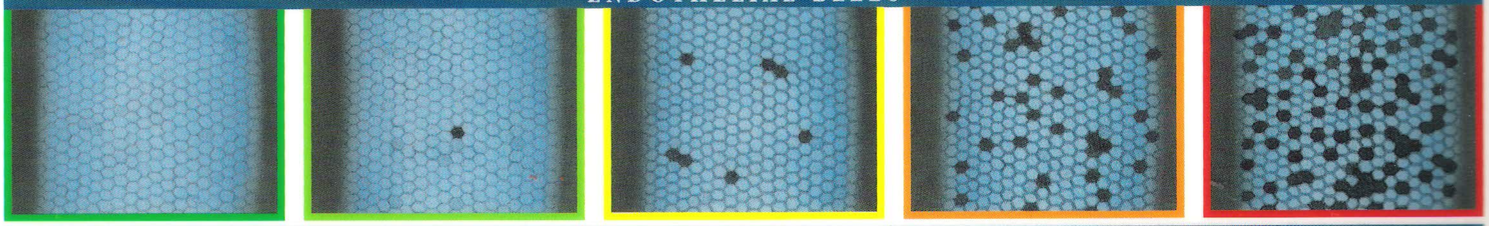

CORNEAL DISTORTION

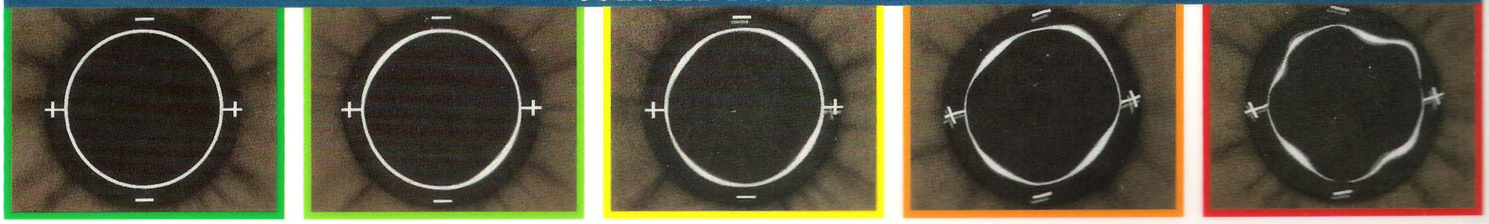

Devised by Professor Nathan Efron and illustrated by Terry R. Tarrant. Millennium Edition. January 1, 2000
Supplement to the book *Contact Lens Complications* by N. Efron published by Butterworth-Heinemann/Optician, 1999, ISBN 0 7506 0582 0

Sponsored by HYDRON™
